# Strain-mediated electric-field control of exchange bias in a Co_90_Fe_10_/BiFeO_3_/SrRuO_3_/PMN-PT heterostructure

**DOI:** 10.1038/srep08905

**Published:** 2015-03-10

**Authors:** S. Z. Wu, J. Miao, X. G. Xu, W. Yan, R. Reeve, X. H. Zhang, Y. Jiang

**Affiliations:** 1 State Key Laboratory for Advanced Metals and Materials, School of Materials Science and Engineering, University of Science and Technology Beijing, Beijing 100083, China; 2 State Key Laboratory of Superlattices and Microstructures, Institute of Semiconductors, Chinese Academy of Sciences, P.O. Box 912, Beijing 100083, China; 3 Institut für Physik, Johannes Gutenberg-Universität Mainz, 55099 Mainz, Germany

## Abstract

The electric-field (E-field) controlled exchange bias (EB) in a Co_90_Fe_10_/BiFeO_3_ (BFO)/SrRuO_3_/PMN-PT heterostructure has been investigated under different tensile strain states. The in-plane tensile strain of the BFO film is changed from +0.52% to +0.43% as a result of external E-field applied to the PMN-PT substrate. An obvious change of EB by the control of non-volatile strain has been observed. A magnetization reversal driven by E-field has been observed in the absence of magnetic field. Our results indicate that a reversible non-volatile E-field control of a ferromagnetic layer through strain modulated multiferroic BFO could be achieved at room temperature.

Multiferroics combining ferromagnetic and ferroelectric properties has been attracted a great amount of interest and continue to stimulate new research activities^1^. BiFeO_3_ (BFO) has outstanding ferroelectric properties and G-type antiferromagnetic ordering in the bulk^2^. BFO has also a relatively high ferroelectric Curie temperature (T_C_ ~ 1100 K), high antiferromagnetic Neel temperature (T_N_ ~ 640 K), high remnant polarization (2P_r_ ~ 110 μC/cm^2^), and low temperature crystallization^3^,^4^. Moreover, BFO is the only known multiferroic material which shows both ferroelectric and magnetic ordering above room temperature^5^. However, since BFO has only a low saturation magnetization due to the canted antiferromagnet spin structure^6^, it is difficult to exploit its multiferroic properties using BFO on its own. To solve this problem, the exchange bias (EB) between BFO and a traditional ferromagnet has been studied, where BFO serves as an antiferromagnet which EB could be controlled by electric-field (E-field)^7^,^8^,^9^. Exchange coupled antiferromagnetic (AFM)/ferromagnetic (FM) bilayers have been widely used in spintronics devices. The E-field control of unidirectional exchange coupling in the AFM/FM heterostructures could lead to a 180 degrees magnetization switching, which presents great significance for information storage such as magnetoelectric random access memories^10^,^11^,^12^,^13^.

Many groups have obtained encouraging results on the E-field control of BFO films^14^,^15^,^16^, however most works on the control of EB by E-field are usually irreversible at room temperature^17^. When directly applying an E-field to a BFO film, the ferroelectric domain walls of the film as the main source of exchange coupling were changed irreversibly^18^. The leakage current across BFO will also increase the difficulty to the application of the E-field to BFO^19^. In this paper, we try to solve this problem by means of strain engineering. Recently, strain engineering^20^ has been used for tuning various properties of perovskite oxide thin films^21^,^22^,^23^. Since the perovskite films tend to grow epitaxially on each other with a slight lattice mismatch^24^, the lattice strain will be generated at the interface of films. Perovskite structures are sensitive to the lattice strain. There are many research works on the changes of the ferroelectric properties for strained BFO films^25^,^26^. However the study on the antiferromagnetic properties of BFO has not received much attentions so far. D. Sando et al.^27^ reported that BFO films deposited on different substrates can produce different kinds of strain from compressive to tensile. The BFO antiferromagnetic magnetization changed with the different strain conditions. In the case of high compressive strain (about −1.7%) and high tensile strain (about +0.5%), the BFO antiferromagnetic moment changes from the cycloidal arrangement to the collinear one. Therefore the EB effect under different strains will also change accordingly. In addition, most work focused on the compressive strain of BFO due to the room temperature phase transition^28^, but less efforts was put on the tensile strain regime.

In order to check the effects of the substrate-induced strain, it is necessary to investigate the properties of thin films with the same heterostructure in order to get rid of some extrinsic effects induced by different types of substrate, growth modes and atomic termination of substrate surface^29^. In this paper, Pb(Mg_1/3_Nb_2/3_)O_3_ (PMN)-PbTiO_3_-(PT) substrates are used to adjust the strain of BFO films by an E-field. The BFO films epitaxially deposited on PMN-PT single crystal substrates allow a strain control from about +0.36% to +0.51%^30^,^31^. At around +0.5% tensile strain, the antiferromagnetic spin state of BFO changes from cycloid to in-plane and the EB shows a significant change between two antiferromagnetic states^27^. By adjusting the tensile strain of the BFO film and choosing appropriate exchange coupling materials, the transition between the two EB states is probably reversible. This transition of EB could realize a reversible 180 degrees magnetization non-volatile switching at room temperature under certain circumstances^32^, which opening up a new possibility for achieving the E-field control devices.

## Results

**Figure 1:**
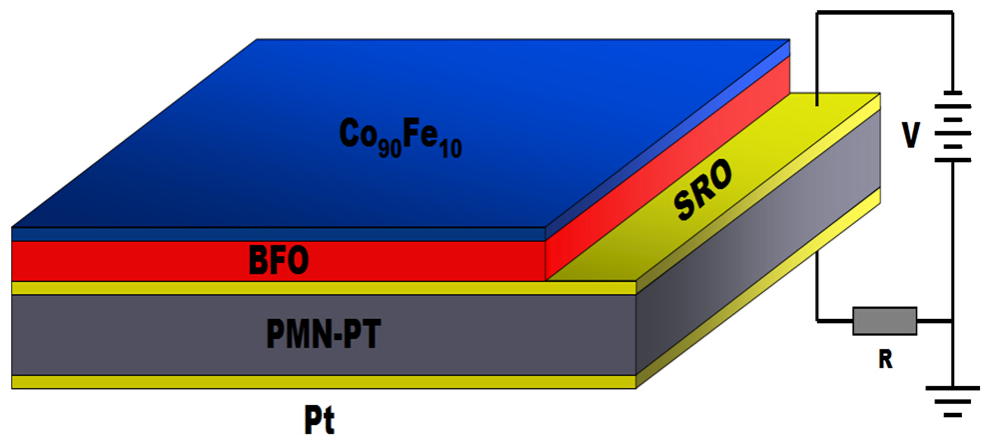
Schematic diagram of the structure Co_90_Fe_10_ (5 nm)/BFO (70 nm)/SRO (5 nm)/PMN-PT/Pt. The polarization voltage was applied between the SRO and Pt electrode in order to avoid directly applying an electric field to the BFO layer.

**Figure 2:**
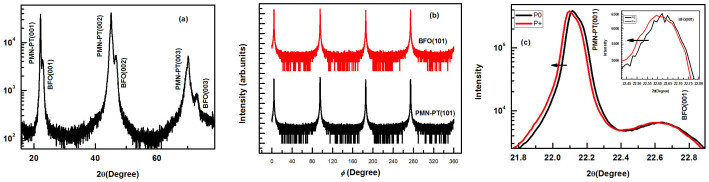
(a) X-ray diffraction pattern of the BFO/SRO/PMN-PT structure. (b) XRD *ϕ* scans of the BFO (101) and PMN-PT (101) reflections. (c) The XRD patterns of the (001) reflections for different polarization states. The peaks of BFO (001) are enlarged in the inset of Fig. 2(c).

Figure 1 shows the schematic diagram of the Co_90_Fe_10_/BFO/SrRuO_3_/PMN-PT sample. Figure 2(a) shows the X-ray diffraction (XRD) pattern of the BFO/SrRuO_3_/PMN-PT structure, revealing the BFO film is highly (001)-oriented. XRD *ϕ*-scans of the BFO (101) and PMN-PT (101) reflections are also performed, as shown in Fig. 2(b). A four-fold symmetry is seen for both the BFO film and the PMN-PT substrate, which indicates a cube-on-cube epitaxial growth of the BFO film on the PMN-PT. The XRD patterns of the (001) reflections for different polarization states are shown in Fig. 2(c), with the peaks of BFO (001) amplified in the inset. The black line corresponds to the PMN-PT substrate in the unpoled state (denoted by P0). The c-axis lattice constant of the film calculated from the BFO (001) reflection is 3.925 Å. That value is smaller than that of the BFO bulk material (c ~ 3.965 Å)^19^, indicating that the film is subjected to an out-of-plane compressive strain (~−1.01%) and an in-plane tensile strain. It is consistent with the fact that the lattice constants of the BFO bulk material are smaller than those of the PMN-PT substrate (a ≈ b ≈ c ~ 4.02 Å)^29^. The in-plane tensile strain can be calculated based on the formula
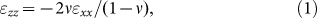
*ε_zz_* is the out-of-plane strain; *ε_xx_* is the in-plane strain; *v* is the Poisson's ratio, assuming approximate volume preserving distortion^29^. The Poisson's ratio *v* of the BFO film is about 0.49^33^. According to Equation 1, the in-plane tensile strain *ε_xx_* is about +0.52%. The red line in Fig. 2 (c) shows the XRD pattern of the structure after applying a +8 kV/cm polarization voltage (denoted by P+). The peaks of BFO film is shift to left and the c-axis lattice constant of BFO increases to 3.932 Å, indicating that the in-plane tensile strain is reduced to +0.43%. This result demonstrates that applying a voltage to the PMN-PT piezoelectric crystal can effectively change the lattice strain of BFO film.

**Figure 3:**
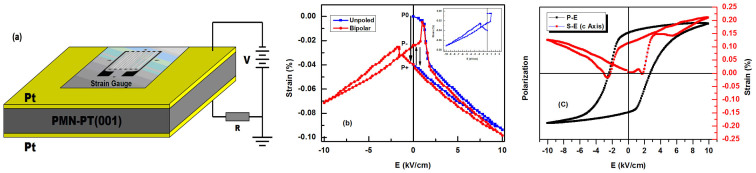
(a) Schematic diagram of using a resistance strain gauge to measure the in-plane strain-electric field (S–E) behavior of the substrate. (b) The red line is the in-plane bipolar S–E curve of the PMN-PT (001). The blue lines in (b) and inset show the typical unipolar S–E curves of the substrate. (c) P–E curve and the out of plane S–E curve of the PMN-PT (001) substrate.

To acquire more detailed strain switching information, a resistance strain gauge was applied to measure the in-plane strain-electric field (S–E) behavior of the PMN-PT (001) substrate. The schematic diagram and measurement results are shown in Figs. 3 (a) and (b), respectively. The blue curve in Fig. 3 (b) is the polar curve of the unpoled substrate, which corresponds to the switch from P0 to P+, with the remnant strain clearly changing during the polarized process. A TF Analyzer 1000 system was also employed to measure the P–E and the out of plane S–E curves of the substrate, as shown in Fig. 3 (c). The red curve is the bipolar S–E curve after the substrate has been poled, which shows a typical asymmetric butterfly shape due to an internal field in the PMN-PT crystal induced by the defects^34^,^35^. With increasing the polarization voltage, the substrate produces a large in-plane compressive strain, but the strain can not be retained well at remanance after the voltage is removed. The inset of Fig. 3 (b) shows that the strain state can be driven back to the position near P0 state by using accurate unipolar control. This unipolar strain modulation is difficult to find the switching point and shows a slow strain relaxation process in our experiment. However, it should be noticed that the remnant strains at zero-field after positive (P+ state) or negative (P- state) polarization was different because of the asymmetric butterfly shape. We can therefore attempt a reversible non-volatile bipolar strain modulation by using this feature.

**Figure 4:**
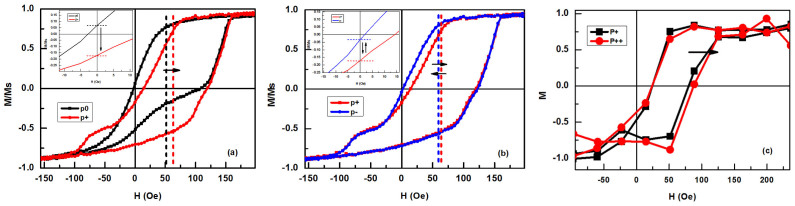
(a) M–H loops of exchange bias (EB) and E-field control effect from P0 to P+, the situation near zero field is amplified in the inset. (b) The M–H curve switching between P+ and P- states which are multi-times reversible, the situation near zero field is amplified in the inset. (c) The M–H curve switching between the polarization P+ state to the P++ state (by applying a +8 kV/cm polarization voltage).

Both obvious EB and E-field control effect have been observed in the sample, as shown in Fig. 4 (a). The EB in one of our samples is about 53 Oe in the P0 polarization state. In the state of P+, the M–H curve moves to right and the shape is slightly changed, with the EB increasing about 14 Oe to 67 Oe. The curves near zero field are amplified in the inset of Fig. 4 (a). Without a magnetic field, the remnant magnetization switches from positive (P0 state) to negative (P+ state) which reveals that some spins in the FM layer have been reversed. To our knowledge, this is the first observation of a large EB generated by tensile strain in BFO and an obvious EB change by an E-field strain control. In Fig. 4 (b), after applying a −8 kV/cm polarization voltage (denoted by P-), the M–H curve slightly moves back to left and the EB decreases about 5 Oe to 62 Oe. The remnant magnetization polarization also decreases at negative direction, as shown in the inset of Fig. 4 (b). Impressively, by a bipolar electric field control, the M–H curve switched between the P+ and P- states is multi-times reversible, but the switch of the remnant magnetization polarization from P0 to P+ cannot been precisely reversed by a unipolar strain modulation. As shown in Fig. 4 (c), the magneto-optical Kerr effect (MOKE) measurement have observed a EB change by control the polarization from P+ state to the state applying +8 kV/cm polarization voltage (denoted by P++), but not as tremendous as that from P0 to P+ state. That result indicates that the relationship between external strain and EB change may not a linear correlation. The specific reason for this phenomenon still needs the further in-depth analysis.

**Figure 5:**
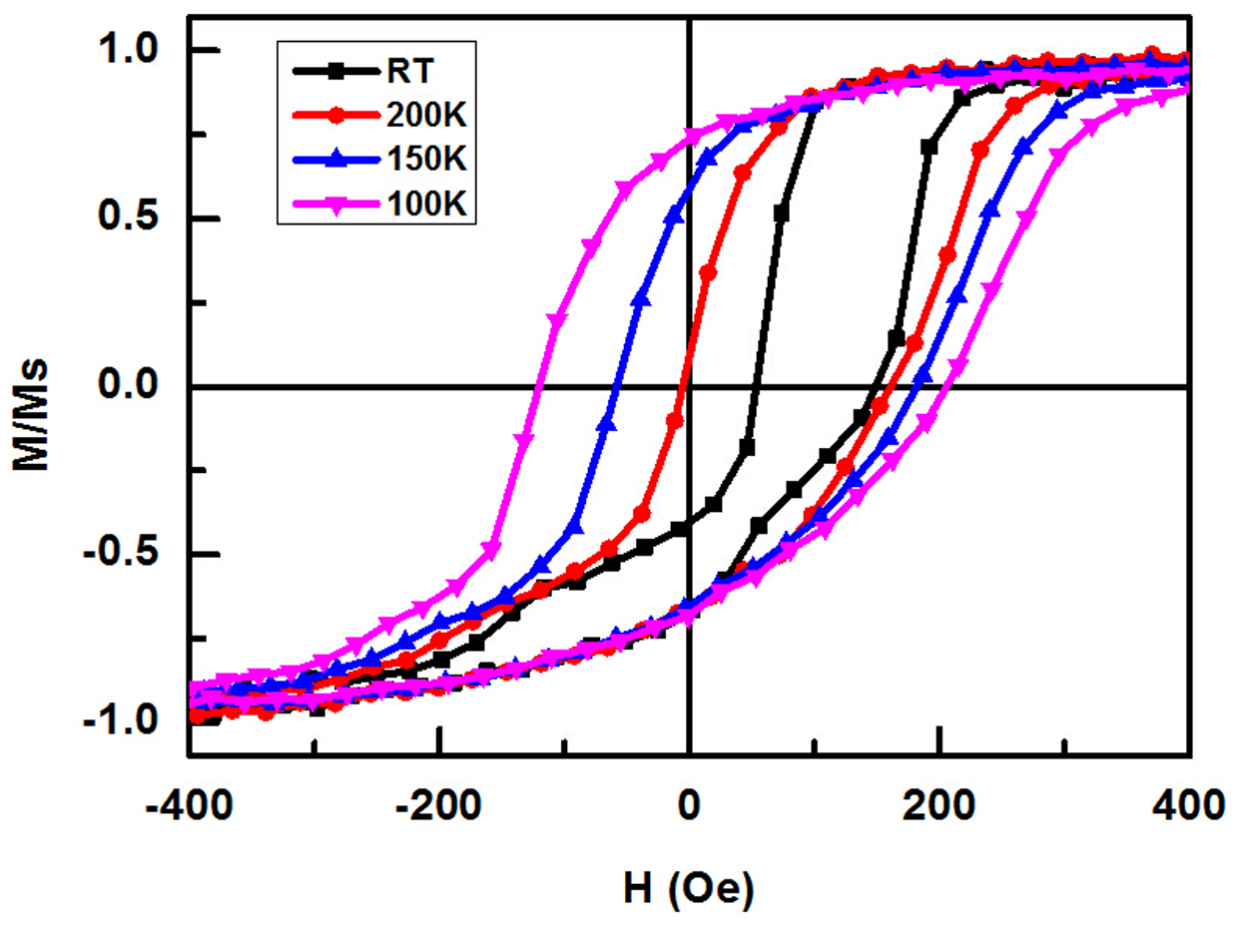
M–H loops of the CoFe/BFO/SRO/PMN-PT heterostructure at different temperatures. With the decreases of the temperature, the coercivity increases significantly and the EB field decreased.

Furthermore, the magnetic behavior of the CoFe/BFO/SRO/PMN-PT heterostructure at different temperatures has been measured. As shown in Fig. 5, with the decreases of the temperature, the coercivity increases significantly and the exchange bias field decreased. Those results are similar to the references report^18^. According to the reference, the coercivity increase with the decrease temperature is due to the enhancing of the exchange interaction of the BFO domains at low temperature. Two typical domain types of BFO can be formed by PLD with different exchange bias properties. Those discrepancies are caused by the different amount of the 109 degrees domain walls. The large EB and magnetic temperature behavior in our samples are similar with one of the domain statues in the reference. Those results indicated the strain-mediated E-field control of exchange bias may also be caused by the changing of the domain walls status. Further research of the strain behavior of different domains seems be interesting.

## Discussion

**Figure 6:**
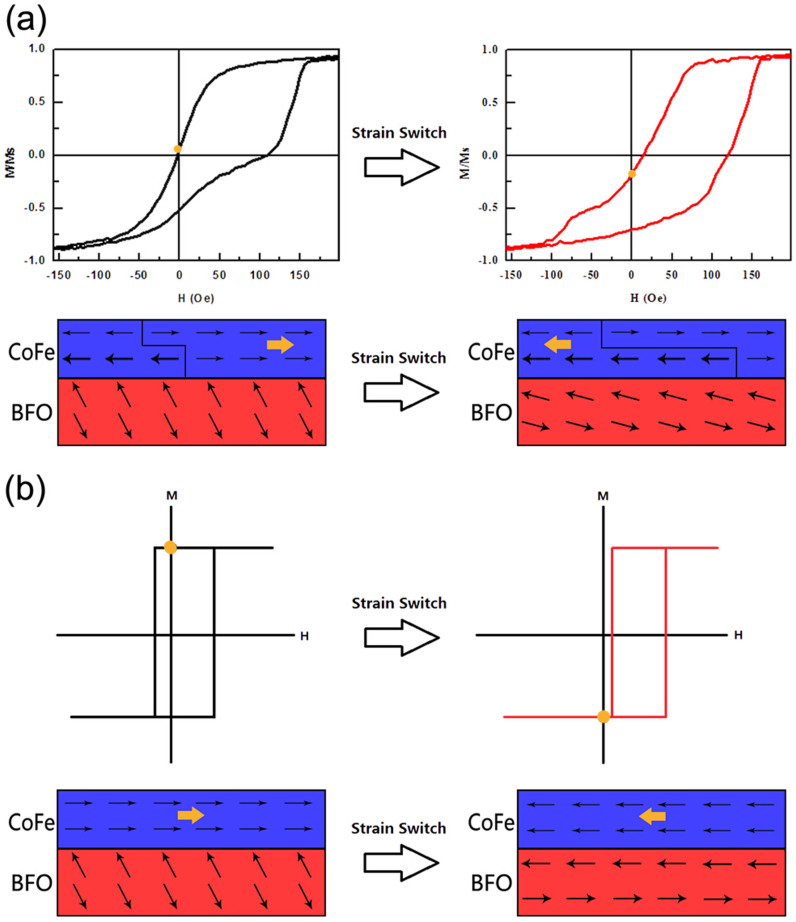
(a) Expected BFO AFM states under the control of strain. The sketch shows the situation of interface spins at the yellow dots of the M–H curves. The yellow arrows indicate the remanent polarization partially changed from positive to negative. (b) A possible way to realize a reversible room temperature non-volatile near 180 degrees magnetization switching in the absence of magnetic field.

According to Sando et al.^27^, antiferromagnetic Fe^3+^ spins point at an angle to the normal direction in the unstrained films. The magnetic states tend to be in-plane cycloid under low tensile strain with the spins pointing along [110] direction and nearly out-of-plane collinear with an in-plane component direction at high tensile strain. The pronounced change of exchange bias observed in this experiment may be caused by the out-of-plane and the in-plane AFM orientation change. According to this theory, at a tensile strain of near 0.5%, the BFO AFM state was changed from the out-of-plane collinear in a high tensile strain to the in-plane cycloid in a low tensile strain. In the P0 state in CoFe/BFO/SRO structure, the in-plane tensile strain of the BFO film is about 0.52% with the AFM state tends to lying in collinear direction and the magnetic polarization direction tends to out-of-plane with an in-plane component direction, which decreases the in-plane EB effect. While in the P+ state, the in-plane tensile strain decreases to 0.43%, the AFM state of BFO is changed into the in-plane [110] cycloid direction, which leads to an in-plane and out-of-plane AFM orientation change and enhances in-plane EB effect. Consequently we can observe the signal switch of the remnant magnetization polarization. Figure 6 (a) schematically shows these expected BFO AFM states under the control of strain. It may explain for the observed large change of EB. Conversely, under the P- state, the in-plane tensile strain increases and the AFM spins tend to out-of-plane more easily than in the P+ state. As a result, we observed a slight decrease of EB.

In addition, it is noteworthy that the modulation of strain in PMN-PT substrates not only affects the AFM state in BFO, but also affects the FM layer as well, such as changing its coercivity or easy axis orientation^36^. For comparison, the Co_90_Fe_10_ film has been directly grown on a SRO/PMN-PT heterostructure. After an applied electric field on the substrate, the M–H curves of Co_90_Fe_10_ show slight difference. However, the strain driven effect of CoFe cannot be totally excluded in this heterostructure. We infer that this effect is one of the minor factors which lead to the large EB change in our samples. Moreover, by choosing appropriate strong anisotropy FM materials, these two effects might be combined to enhance the E-field control of the EB effect^32^.

Encouragingly, our results reveal a possibility to realize a reversible non-volatile near 180 degrees magnetization switch in the absence of magnetic field at room temperature. Figure 6 (b) gives a conceivable way to realize it by adjusting an non-volatile strain on the substrate, choosing appropriate FM materials, and inducing high EB effect by controlling the domains and the easy axis of BFO film^37^.

In summary, the non-volatile tensile strain states of the BFO film have been formed on the PMN-PT substrate. The strain-induced room temperature E-field control of EB has been studied in our samples. A notable change of EB from 53 Oe (at +0.52% tensile strain) to 67 Oe (at +0.43% tensile strain) was observed. We observed the E-field induced magnetization reversal in the absence of magnetic field. We accordingly propose that it is possible to realize near 180 degrees reversible switching of FM layer by E-Field control at room temperature through adjusting the tensile strain of the BFO film and choosing appropriate FM materials.

## Methods

The thin films of BFO (70 nm)/SrRuO_3_ (5 nm) were epitaxially grown on a (001)-oriented (1 − x)PMN-xPT (x = 0.3, PMN-PT with 30% of PT) single crystal substrate by pulsed laser deposition. The BFO thickness was set to 70 nm to avoid structural relaxation. The temperature of the substrate was kept at 650°C and the oxygen pressure was 10 Pa. After the deposition, the as-grown films were *in situ* annealed in pure oxygen of 200 Pa for 30 minutes and then slowly cooled down to room temperature. The crystalline quality of the films was carefully examined by XRD. After that, a 5 nm Co_90_Fe_10_ film was *in situ* grown on the BFO film by magnetron sputtering, applying an in-plane magnetic field along [100] direction about 200 Oe to induce the easy axis. The backside of the substrate was coated with Pt to act as the bottom electrode. The magnetic hysteresis loops of the sample were measured by an alternating gradient magnetometer, physical property measurement system and MOKE. A high-voltage power supply was employed to apply a polarization voltage between the SRO film and Pt electrode, and a 20 M Ohm resistor was added to protect the circuit.

## Author Contributions

S.Z.W. conceived and designed the study. W.Y. and X.H.Z. took the MOKE measurement. Prof. Y.J. and Prof. J.M. planned and supervised the study. R.R. and X.G.X. gave out the amendments for the manuscript. All authors contributed to the scientific discussions.
